# Preoperative fasting and the risk of pulmonary aspiration—a narrative review of historical concepts, physiological effects, and new perspectives

**DOI:** 10.1016/j.bjao.2024.100282

**Published:** 2024-05-05

**Authors:** Anne Rüggeberg, Patrick Meybohm, Eike A. Nickel

**Affiliations:** 1 Department of Anaesthesiology and Pain Therapy, Helios Klinikum Emil von Behring, Berlin, Germany; 2 Department of Anaesthesiology, Intensive Care, Emergency and Pain Medicine, University Hospital Würzburg, Würzburg, Germany

**Keywords:** 2 h fasting, clear fluid, clear liquid, drink until call, liberal fasting, preoperative fasting, Sip Til Send, unrestricted drinking

## Abstract

In the early days of anaesthesia, the fasting period for liquids was kept short. By the mid-20th century ‘nil by mouth after midnight’ had become routine as the principles of the management of ‘full stomach’ emergencies were extended to include elective healthy patients. Back then, no distinction was made between the withholding of liquids and solids. Towards the end of the last century, recommendations of professional anaesthesiology bodies began to reduce the fasting time of clear liquids to 2 h. This reduction in fasting time was based on the understanding that gastric emptying of clear liquids is rapid, exponential, and proportional to the current filling state of the stomach. Furthermore, there was no evidence of a link between drinking clear liquids and the risk of aspiration. Indeed, most instances of aspiration are caused by failure to identify aspiration risk factors and adjust the anaesthetic technique accordingly. In contrast, long periods of liquid withdrawal cause discomfort and may also lead to serious postoperative complications. Despite this, more than two decades after the introduction of the 2 h limit, patients still fast for a median of up to 12 h before anaesthesia, mainly because of organisational issues. Therefore, some hospitals have decided to allow patients to drink clear liquids within 2 h of induction of anaesthesia. Well-designed clinical trials should investigate whether these concepts are safe in patients scheduled for anaesthesia or procedural sedation, focusing on both aspiration risk and complications of prolonged fasting.

The Helsinki Declaration on Patient Safety in Anaesthesiology states: ‘Patients have a right to expect to be safe and protected from harm during their medical care and anaesthesiology has a key role to play improving patient safety perioperatively’.^1^ Accordingly, the European Society for Clinical Nutrition and Metabolism (ESPEN) guideline ‘Clinical Nutrition in Surgery’ recommends avoidance of long periods of preoperative fasting^2^ and ‘Choosing Wisely in paediatric anaesthesia’ demands shorter real fasting times.^3^ This is nothing new. Even in the early days of anaesthesia, it was highlighted that preoperative fasting for several hours would exacerbate existing states of exhaustion. Nevertheless, in the decades that followed, ‘nil by mouth after midnight’ became the norm, even for healthy elective patients with no risk factors for aspiration.

## How it all began

When anaesthesia was in its infancy in the mid-19th century, preoperative fasting was recommended to minimise the discomfort of nausea and vomiting.^4^ In the case of 15-yr-old Hannah Greener, who died under ether anaesthesia in 1848, it remained unclear whether an overdose of ether or aspiration of orally administered brandy led to death.^5^ The first proven fatal aspiration was published in 1862. As early as 1853, a soldier in Burma vomited during surgery for a bullet wound in the leg and died shortly afterwards. The autopsy showed that the trachea was full of vomitus.^4^

The British surgeon Sir Joseph Lister published simple, practical guidelines for fasting in 1883, and was the first to distinguish between solids and clear liquids: ‘While it is desirable that there should be no solid matter in the stomach when chloroform is administered, it will be found very salutary to give a cup of tea or beef-tea about two hours before’.^4^ As ‘several hours of preoperative fasting aggravate existing states of exhaustion’, tea with red wine or cognac up to 45 min before induction of anaesthesia was recommended at the beginning of the 20th century, especially for alcoholics.^6^

### Mendelson's ‘Nil by mouth’

In 1946, the obstetrician Mendelson retrospectively described 66 cases of aspiration of gastric contents during anaesthesia in 44 016 pregnant women, an incidence of 0.15%. Two patients died, both from complete airway obstruction by aspirated solid food. There were no deaths among the 40 patients who aspirated liquid stomach contents; they developed asthma-like symptoms that soon subsided. Mendelson showed that hydrochloric acid or non-neutralised liquid human vomit instilled into the lungs of anaesthetised rabbits caused immediate cyanosis, respiratory distress, and changes on chest radiographs similar to those seen in patients with liquid aspiration.^7^ Mendelson recommended ‘withholding oral nutrition during delivery to prevent aspiration’ and made no distinction between clear liquids and solids. In the following decades, the fear of Mendelson's syndrome led to the concept of ‘nil by mouth after midnight’. Unfortunately, this applied not only to high-risk patients, but also to healthy patients without risk factors undergoing elective surgery.^4^

### Roberts' threshold for the risk of aspiration

The ‘nil by mouth after midnight’ concept was reinforced when 25 ml in the stomach, present in half of all healthy fasting patients, was adopted as a surrogate marker for a high risk of aspiration.^4^ Citing his own unpublished data, Roberts stated in 1974: ‘Our preliminary work in the rhesus monkey suggests that 0.4 ml kg^−1^ body weight is the maximum acid aspirate which does not produce significant changes in the lungs’.^8^ In 1980, Roberts described that the gastric acid applied directly to the left lung via a tracheostomy tube in this study had a pH of 1.26.^9^ The number of animals used remained unclear. Roberts went on to write in 1974 that as 0.4 ml kg^−1^ body weight ‘is equivalent to about 25 ml in the adult human female, we have arbitrarily defined the patient at risk as that patient with at least 25 ml of gastric juice with a pH below 2.5 in the stomach’.^8^ In the following decades, this threshold was referred to as the ‘critical gastric volume in relation to the risk of aspiration’.

However, fasting elective patients very often exceed the threshold of 0.4 ml kg^−1^ for gastric residual volume.^10^^,^^11^ Recent studies have arbitrarily set the threshold for distinguishing between an empty and full stomach at 1.5 ml kg^−1^,^11^^,^^12^ which corresponds to the 95th to 97th percentile of gastric volume in healthy fasting adults. However, the threshold gastric volume that could lead to an increased risk of regurgitation is probably much higher, exceeding 8 ml kg^−1^ in animal studies.^13^ To the best of our knowledge, there are no corresponding studies in humans.^12^

## Learning points

• Historically, fasting times have been arbitrarily set with the aim of preventing aspiration.
• For decades, no differentiation has been made between clear liquids and solids (nothing by mouth after midnight).

## Gastric emptying

**Fig 1:**
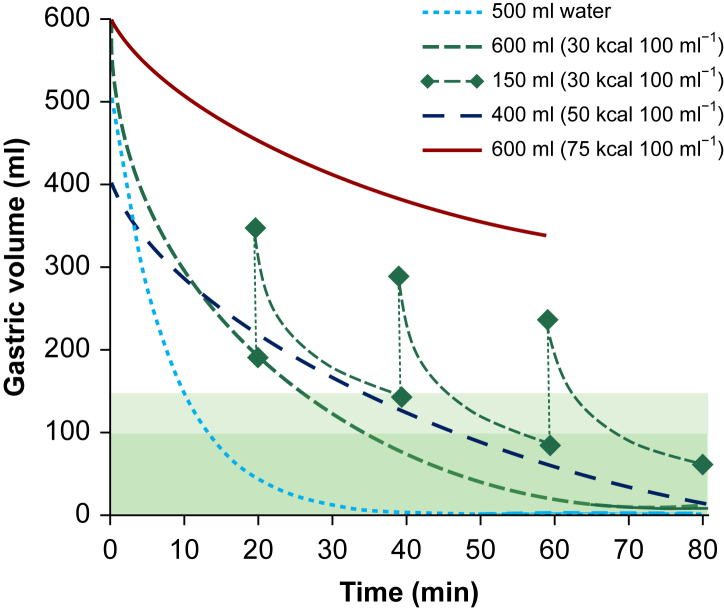
Gastric emptying after drinking water and clear carbohydrate liquids with three different calorie contents. The green dashed lines between the diamonds represent repeated fluid intake of 150 ml. The range of normal gastric residual volumes is indicated in green (according to data from^11^^,^^14^, ^15^, ^16^, ^17^, ^18^).

Gastric emptying of clear liquids is mainly affected by the volume of fluid in the stomach and the energy density of the liquid (Fig. 1). Gastric emptying exponentially increases as fluid volume in the stomach increases and is also proportional to the rate of filling.^14^^,^^15^^,^^19^, ^20^, ^21^, ^22^ Drinking clear liquids therefore speeds up the emptying of the stomach.^15^^,^^23^

Secondly, the higher the energy density of the liquid, the slower the gastric emptying.^16^^,^^21^^,^^23^, ^24^, ^25^ Receptors in the small intestine regulate gastric emptying to about 200 kcal per hour to prevent the intestine from receiving more nutrients than it can absorb.^14^^,^^17^ Therefore, liquid gastric emptying depends mainly on the total caloric content.^18^^,^^26^ There were no significant differences in liquid gastric emptying after drinking equal amounts of either orange juice or milk, as long as both had the same number of calories.^18^

For high-calorie non-clear liquids, calorie and nutrient content affect gastric emptying times.^25^ The stomach empties carbohydrates faster than proteins, and fat stays in the stomach the longest.^27^ Analysis of the area under the curve of the gastric content volume–time profile, which may be a more sensitive measure of gastric emptying kinetics, shows that the area under the curve is greater for a high-fat drink than for an isocaloric high-carbohydrate drink.^25^

In comparison to energy density, osmolality has less of an effect on gastric emptying. Replacing glucose monomers with polymers may increase the rate of gastric emptying, but this osmolality effect is more pronounced at high carbohydrate concentrations.^17^

Importantly, the gastric emptying rate of liquids shows a high inter-subject variability of about 30%, while the intra-subject variability is <10%.^14^ The high variability of gastric emptying after liquid intake is also explained by the fact that the stomach emptying is episodic. Consequently, measuring gastric volumes will result in different data depending on the time points just before or just after episodic emptying.

Clear liquids quickly leave the stomach. There is a functional tunnel along the small curvature of the stomach which allows liquids to pass directly into the duodenum within 10 min, bypassing the greater part of the stomach.^28^ When volunteers drink 500 ml of water, more than half of the water has left the stomach after 10 min, and the stomach is empty after 20 min.^18^ If a subject drinks 600 ml of a carbohydrate drink (30 g L^−1^ glucose), half of the liquid will have left the stomach after 15 min and the stomach will be empty after 30 min. If subjects drink 600 ml of the carbohydrate drink first and then continue drinking 150 ml every 20 min, they will have ingested a total volume of 900 ml in less than an hour, consumed the energy of a small breakfast, and still have an empty stomach.^14^^,^^15^ The gastric emptying time after drinking tea or coffee with milk (up to about one-ﬁfth of the total volume) is comparable to that of water or pure tea.^29^, ^30^, ^31^, ^32^ Trauma, such as fractures of the radius or hip, does not delay gastric emptying of clear liquids,^33^^,^^34^ nor does obesity^35^ or diabetes mellitus.^36^ Gastric emptying is independent of age^37^ and is not affected by carbonated liquids.^38^^,^^39^

In addition to oral liquid intake, 2.5 L of saliva and gastric juice pass through the stomach each day.^40^ Their secretion can increase up to 600 ml h^−1^ in the cephalic secretion phase (triggered, for example, by thoughts of food). Therefore, fasting does not guarantee an empty stomach.

### Gastric emptying of solid food

Differentiating between gastric emptying of solids and liquids is very important. In addition to the digestion and breakdown of food, the stomach also has a reservoir function.^19^ This function does not apply to water and clear liquids with low energy densities, which pass through the stomach quickly, proportionally to the rate of filling.

According to European guidelines, breastfeeding should be encouraged up to 3 h before anaesthesia, milk and light solid foods up to 4 h and solid foods up to 6 h before induction of anaesthesia for children.^41^ In adults, solid food should be prohibited for 6 h before elective surgery.^32^ But American guidelines explicitly point out that both the amount and type of foods ingested have to be considered when determining an appropriate fasting period for solids. A longer fasting time (e.g. 8 h or more) may be needed after fried foods, fatty foods, or meat and for patients with coexisting diseases or conditions that can affect gastric emptying (see below).^42^

## Learning points

• Clear liquids leave the stomach very quickly, with a half-life of 10–15 min for clear liquids that have no calories or low calorie content.
• The amount and type of solid food consumed must be considered when determining a suitable fasting period.

## Risk factors for pulmonary aspiration

Recent studies have defined the normal gastric fluid volume as up to 1.5 ml kg^−1^.^11^ But 1.5 ml kg^−1^ gastric fluid volume represents the upper end of normal baseline gastric secretions. Therefore, with this definition up to 6–13% of patients who have formally fasted will have an increased gastric fluid volume.^11^^,^^43^, ^44^, ^45^, ^46^ Cho and colleagues^47^ found a fasting gastric volume >1.5 ml kg^−1^ in 31% of gynaecological patients and Zhou and colleagues^48^ in 48% of patients with diabetes. However, the incidence of perioperative aspiration is only 0.02–0.04% in retrospective studies^49^^,^^50^ and 0.02–0.07% in elective adults and children in prospective studies.^51^, ^52^, ^53^ Therefore, gastric volume does not appear to be a suitable surrogate marker for the risk of pulmonary aspiration and, in particular, for the risk of pneumonia.

Sonographic evidence of fluid in the stomach is not associated with a higher risk of aspiration. Drinking clear liquids fills the stomach but also increases the compliance of the gastric fundus while intragastric pressure is stable until the gastric volume is 1 L or less.^19^^,^^24^ But if the pressure in the stomach increases, the tone of the lower oesophageal sphincter also increases, so that the barrier pressure between the stomach and oesophagus is maintained.^54^^,^^55^

### Pre-existing conditions

Comorbidities are not independent risk factors for pulmonary aspiration.^50^ Laryngeal incompetence, for example in neuromuscular diseases, leads to dysphagia. In this case, the risk of aspiration is increased only during the swallowing process.

Liquids pass through the oesophagus within 1 s when the body is in an upright position. Even in severe swallowing disorders such as achalasia, gastric emptying is not affected. Reflux disease is a disorder of motility and function of the oesophagus or cardia that does not affect gastric emptying.

Conditions that may be associated with delayed gastric emptying are often cited as reasons for the need for a 2-h liquid fast. But it is often the case that no distinction is made between the gastric emptying of solids and liquids. This distinction is crucial, and is important to understand. Aspiration of clear liquids is rarely of clinical significance, whereas aspiration of solids often leads to serious complications.^56^ However, gastric emptying is usually delayed only for solids. Gastric ultrasound in patients with risk factors for gastroparesis revealed the presence of solid food in the stomach in 12.5% of fasted patients.^57^

In patients with gastroparesis, gastric emptying of clear liquids is usually unaffected, and sometimes even accelerated. One example is diabetic gastroparesis, which primarily affects solids.^58^, ^59^, ^60^ Gastric emptying after carbohydrate drinks is even faster in patients with type 2 diabetes than in those without diabetes.^61^ Similarly, vagotomy results in rapid fluid and delayed solid emptying.^58^ Some drugs, such as semaglutide, delay gastric emptying for solids.^62^ Perioperative semaglutide use was associated with increased residual gastric volume, but in 85.2% of 33 patients, solid content was observed.^62^ Only four patients met the study criteria for an increased gastric fluid volume of >0.8 ml kg^−1^, but this is a normal fluid level.

### Aspiration pneumonia

**Fig 2:**
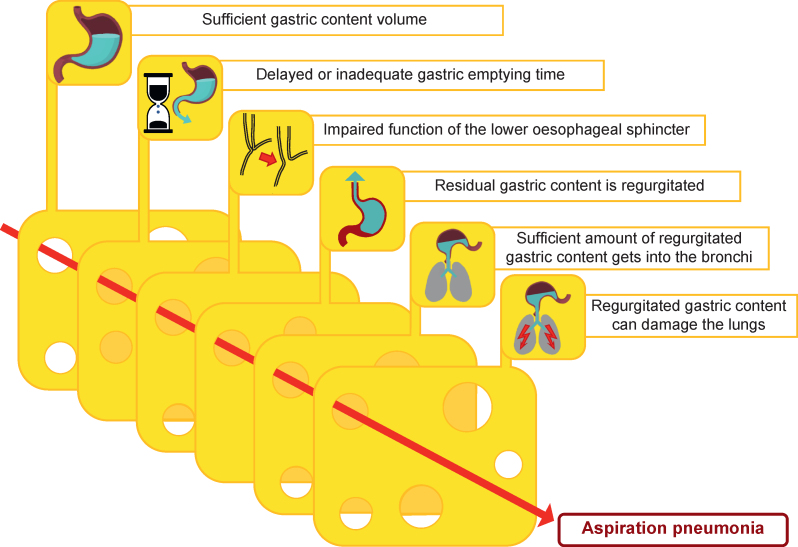
Factors that must coincide for patients to develop aspiration pneumonia.

Based on the aforementioned findings, pulmonary aspiration in elective patients is the result of the coincidence of several factors (Fig. 2):

o A sufficient volume of gastric content must be present.
o Delayed or inadequate gastric emptying time.
o The function of the lower oesophageal sphincter must be impaired or unable to withstand the applied pressure (e.g. reflex reactions of the patient during tracheal intubation under insufficiently deep anaesthesia).
o The residual gastric content must be regurgitated.
o Sufficient regurgitated gastric content must reach the bronchi.
o The regurgitated gastric content must be harmful to the lungs.

The presence or occurrence of individual factors does not lead to aspiration pneumonia.

### Prevention of aspiration

Most aspiration events are as a result of failure to recognise the risk factors for aspiration (see Table 1) and to adjust the anaesthetic technique accordingly.^63^^,^^64^ Therefore, good preoperative patient assessment and staff training with adaptation of anaesthetic techniques can prevent aspiration. Particular attention should be paid to the indication and correct performance of a rapid sequence induction. In an online survey, 1921 members of the European Society of Anaesthesiology from 56 countries were asked about their clinical practice of rapid sequence induction.^65^ The majority (61.7%) of the respondents preoxygenated patients with O_2_ 100% for 3 min and 65.9% administered opioids during rapid sequence induction. In all patients where rapid sequence induction was indicated, the Sellick manoeuvre was used by 38.5% and was never used by 37.4% of the respondents. The remaining respondents only performed the Sellick manoeuvre in certain patient groups. First-line medications for haemodynamically stable adult patients were propofol (90.6%) and suxamethonium (56.0%). Manual ventilation (inspiratory pressure <12 cm H_2_O) was used by 35.5% of the respondents.

**Table 1:** Predisposing factors for pulmonary aspiration. Data from.^50^^,^^63^^,^^67^, ^68^, ^69^, ^70^

Full stomach	Recent eating
	Ileus (paralytic, non-paralytic)
	Pregnancy
	Delayed gastric emptying (traumatised patients, pyloric spasm)
	Gastric hypersecretion (pain, stress)
	Advanced chronic disease resulting in gastroparesis (diabetes mellitus/chronic kidney disease/neuromuscular disorders)
Oesophageal sphincter	Severe reﬂux disease
	Oesophageal disorders: Zenker's diverticulum, strictures
	Previous oesophageal-gastric surgery
	Morbid obesity
Laryngeal reﬂexes	Inadequate depth of anaesthesia
	Traumatic brain injury, cerebral infarction
	Neuromuscular disorders
Others	Emergency surgery
	Difficult airway
	Inadequate use of first generation supraglottic airway devices

An international survey of 10 003 anaesthetists from 141 countries demonstrated that preferences for positioning (head-up or head-down), nasogastric tube use, and cricoid force application during rapid sequence intubation vary substantially, but were routinely performed for a hypothetical patient with intestinal obstruction.^66^ When anaesthetists were asked to identify the most important learning point from their experience with aspiration, their response was to address gastric decompression before anaesthesia. This includes placing a nasogastric tube if not already present, applying suction through it, administering a small amount of saline to unblock a potentially obstructed tube, and changing the patient position on the operating table to facilitate gastric emptying. For more details on rapid sequence induction, see the review by Collins and O'Sullivan.^71^

However, there is a lack of evidence from randomised controlled trials for many of the interventions that constitute rapid sequence induction.^65^^,^^72^ In particular, cricoid pressure is controversial, but a large, controlled, randomised trial from France found no advantage in terms of pneumonia and mortality in the cricoid pressure group, but a significantly higher rate of more difficult laryngoscopy.^73^ Contrary to Sellick's assumption,^74^ more recent sonographic studies have shown that the oesophagus is displaced laterally rather than dorsally to the cervical spine when cricoid pressure is applied.^75^ However, prophylactic strategies to prevent aspiration also carry potential risks of side-effects (e.g. rapid sequence induction with unexpected difficult intubation); therefore a critical appraisal of the risk factors is needed.^76^

There is no evidence of a link between drinking clear liquids and the risk of aspiration in elective patients.^12^ Of course, emergency patients with an indication for rapid sequence induction must remain fasting from the time of diagnosis, even for clear liquids. In cases of doubt, gastric ultrasound can be used as a ‘point-of-care’ procedure when considering whether to place a gastric tube before induction of anaesthesia and perform a controlled ‘rapid sequence induction’. A scientific evaluation of this topic is expected in the recommendations of new guidelines on perioperative fasting. The usefulness of ultrasound in assessing gastric contents and the associated risk of aspiration is one of the main topics of this upcoming guideline.^77^ Where possible, performing surgery with regional anaesthesia may be an alternative.

## Learning points

• Most cases of aspirations are associated with failure to recognise risk factors for aspiration and failure to adjust the anaesthetic technique accordingly.
• Gastroparesis mainly delays the gastric emptying of solids, but not liquids.

## Prolonged liquid fasting

International guidelines have recommended 2-h clear liquid fasting before induction of anaesthesia in adults.^32^^,^^42^^,^^67^, ^78^, ^79^ In clinical practice, however, adult patients fast for a median of 9–12 h.^11^^,^^80^, ^81^, ^82^, ^83^, ^84^ Prolonged liquid deprivation not only causes discomfort such as thirst, anxiety, fatigue, and postoperative nausea,^80^^,^^82^^,^^85^, ^86^, ^87^, ^88^, ^89^, ^90^, ^91^, ^92^, ^93^, ^94^, ^95^, ^96^, ^97^, ^98^, ^99^, ^100^ but can also lead to serious postoperative complications. The duration of preoperative liquid fasting is a modifiable precipitating factor of delirium in the post-anaesthesia recovery room (odds ratio 2.69) and on the ward (odds ratio 10.57).^101^ Preoperative dehydration quadruples the number of postoperative complications after hip fracture, especially neurological, cardiovascular, renal, or respiratory problems.^102^ Dehydrated tumour nephrectomy patients have an increased risk of postoperative renal failure.^103^ Preoperative oral rehydration solution supplementation significantly reduced post-spinal transient ischaemic electrocardiographic changes in older patients.^104^

In adults, prolonged fasting resulted in perioperative hyperglycaemia, insulin resistance, increased interleukin-6 levels, urinary ketone excretion, impaired cardiac output, and psychosomatic status, and decreased muscle strength compared with drinking carbohydrate drinks 2 h before induction of anaesthesia.^91^^,^^93^^,^^95^^,^^98^^,^^105^, ^106^, ^107^, ^108^, ^109^, ^110^, ^111^, ^112^, ^113^, ^114^ Perioperative hyperglycaemia is associated with an increased risk of infection, reoperation, and mortality in patients undergoing visceral surgery.^115^ Patients who drink coffee regularly have an increased risk of postoperative caffeine withdrawal headache.^116^

During long periods of fasting, children also experience thirst, hunger, anxiety, vomiting, and pain.^117^, ^118^, ^119^ Prolonged preoperative fasting may be a risk factor for postoperative emergence delirium in children.^120^^,^^121^ Optimised preoperative fasting management reduces fasting time, decreases ketone body concentration, and helps to stabilise mean arterial blood pressure during induction of anaesthesia in children.^122^^,^^123^

Up to 13% of patients deliberately ignore fasting recommendations for eating or drinking in order to avoid fasting for several hours.^124^, ^125^, ^126^, ^127^, ^128^ Some patients would lie about how long they had fasted if they were told that their operation would be delayed or cancelled because they had eaten or drunk something.^125^^,^^127^^,^^128^ Approximately 2–3% of patients do not adhere to the fasting rules for solids, putting themselves at serious risk. Although pulmonary aspiration of clear liquids is rarely of clinical significance, aspiration of solid food often leads to serious complications.^56^

### Reasons for prolonged liquid fasting

**Fig 3:**
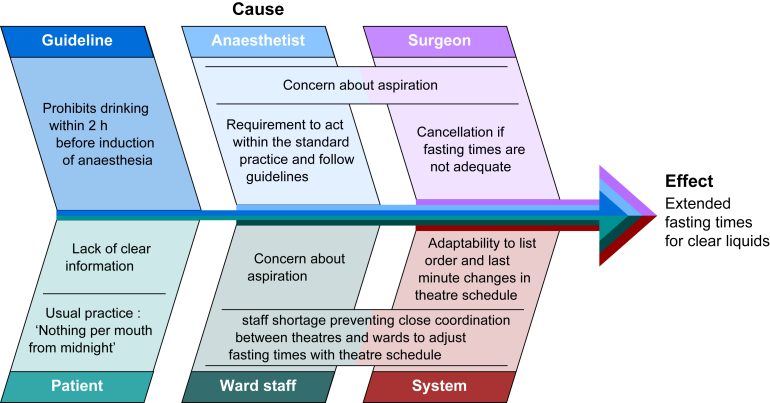
Ishikawa diagram summarising the main reasons for prolonged liquid fasting.

The main reasons for prolonged liquid fasting are summarised in the Ishikawa diagram in Figure 3. Systemic reasons focussed on problems in the organisational implementation of guidelines. Human reasons are based on concerns about aspiration if the 2 h limit is not met. To better understand the latter, knowledge of the risk factors for aspiration in elective patients and knowledge of gastric emptying—pathophysiology and clinical studies—are required (see above).

Quality improvement measures to implement the 2 h liquid fasting have not yet resulted in liquid withdrawal times in the range recommended by guidelines.^129^, ^130^, ^131^, ^132^, ^133^ In a survey of Indian anaesthetists, only 20% reported following the ASA guideline.^134^ In an international survey, only about half of the anaesthetists recommended that patients continue to drink clear liquids up to 2 h before surgery.^135^ The main reasons given for these discrepancies were uncertainty of scheduling of the surgical procedures and failure to implement the guideline.^134^^,^^135^ In an operating theatre the exact time to start anaesthesia is often unpredictable and affected by short-term re-scheduling.^56^ Up to 12–16% of operations have been cancelled on the day that they were scheduled, mainly because other operations took longer than planned, emergency cases overwhelmed theatre capacity, or operations were cancelled because of limited personal or material resources or patient reasons.^136^^,^^137^ In fact, neither patients nor hospital staff know exactly what time intake of clear liquid should be stopped.^56^ Close communication between the operating theatre and ward would be necessary but is often limited because of the workload, staff shortages, and surgeons' concerns that the anaesthesia department may refuse to anaesthetise the patient if the liquid fasting time was too short.^56^^,^^134^

Giving i.v. fluids does not seem to be a solution. Oral liquid withdrawal in patients who receive i.v. fluids while waiting for surgery is almost twice as long as in patients who do not receive i.v. fluid replacement (12.5 *vs* 6.5 h), and they experience significantly more thirst and thirst-related distress.^138^ However, infusions should be given if they are needed because of pre-existing conditions or other medical reasons.

## Learning points

• Prolonged liquid fasting reduces patient comfort and may lead to serious postoperative complications.
• The main reasons for the failure to implement the recommendation of 2 h are the variable timing of surgical procedures and the (historical) fear of aspiration.

## And today

Current guidelines recommend 2 h of liquid fasting,^32^^,^^42^^,^^78^^,^^79^ which translates into median fasting times of up to 12 h in clinical practice.^80^^,^^83^^,^^129^ Because many anaesthetists refuse to induce anaesthesia in patients who have liquid fasting for <2 h, mostly for medico-legal reasons, significant reductions in prolonged fasting times have not yet been achieved. The lack of implementation of guidelines puts patients at risk in several ways. Reduced wellbeing increases the perioperative stress response, dehydration increases the incidence of severe postoperative complications, and lack of energy intake leads to insulin resistance and, thus, muscle breakdown. Patients put themselves at risk by deliberately breaking the fasting recommendations for solids.

Strict adherence to the recommendation of at least 2 h of liquid withdrawal is therefore incompatible with the recommendation that patients should not fast for >2 h because of the risk of postoperative complications. Fulfilling both requirements of the guidelines—at least 2 h, but not >2 h—appears to be practically impossible, especially for hospitals providing emergency care. To decide which of the two guideline requirements is considered more important, anaesthetists are increasingly carrying out a risk assessment between these two options:

- adherence to the 2-h limit, which in practice usually results in excessively prolonged liquid fasting times or
- allowing shorter liquid fasting times even shorter than 2 h to reduce perioperative complications.

The current adult guidelines only consider studies that investigated liquid fasting times between 2 and 4 h *vs* >4 h, or conclude that drinking up to 2 h before induction of anaesthesia has no effect on, or even reduces, gastric residual volume.^32^^,^^42^ There is neither evidence nor any theoretical pathophysiological explanation for harm after clear liquid fasting for <2 h. Therefore, a recent international multidisciplinary consensus statement recommends that all patients with no or low risk of aspiration should receive unrestricted clear liquids before procedural sedation.^139^

This is why more and more anaesthetists consider concepts such as ‘Sip Til Send’, ‘drink until called’ or ‘unrestricted drinking before surgery’.^42^^,^^52^^,^^83^^,^^85^^,^^140^, ^141^, ^142^

## Learning points

• To date, there is no system that makes it possible to comply with both recommendations of the guidelines—at least 2 h, but not much more than 2 h.
• As a result, an increasing number of hospitals are weighing up the pros and cons of prolonged liquid fasting or of going below the recommendation of 2 h.

## What approaches have individual centres implemented to shorten liquid fasting times?

In paediatric anaesthesia, liberal fasting regimens are much more common than in adult anaesthesia, where there are only a few published approaches to the implementation of shorter fasting times.

### For children

For children, the recommendation for clear liquid fasting was reduced to 1 h in 2022.^41^ The extent to which liberalisation of the preoperative liquid fasting time to the new recommendation of 1 h^41^ will be translated into clinical reality remains to be seen. In a quality management project involving around 16 000 children, the introduction of the 1 h limit only reduced the median liquid fasting time from 9 to 6 h.^143^ Implementation of the 1-h fasting instruction reduced the median effective fasting time for clear fluids to 2.6 h in a prospective, observational, multi-institutional cohort study of 22 766 paediatric anaesthetics.^144^ In the NIKs study, clinics with a ‘drink until call’ regimen had the shortest liquid fasting times of 1.8 h (*n*=4188) compared with clinics with a 6/4/1 regimen (2.5 h, *n*=7163) or a 6/4/2 regimen (3.7 h, *n*=742.)^145^ The Uppsala working group, which has been practising ‘no limitations on clear liquid intake until called to theatre’ in children for >20 yr, reported a median fasting time of 1 h.^49^^,^^146^ Allowing children to drink clear liquids until premedication significantly reduces the actual fasting time from 3.9 h to 48 min.^147^ In none of the studies was a shorter fasting time an independent risk factor for aspiration.^49^^,^^144^^,^^145^^,^^147^

### For adults

To find out what approaches individual centres have taken to reduce fasting times in adults, we conducted a literature search (PubMed) to identify relevant articles. The search terms were: fasting and anaesthesia, language English and date limit was set from year 2020 onwards. We identified 425 articles to assess for relevance. Of these, 148 articles were relevant to preoperative liquid fasting and 14 reported approaches to reduce liquid fasting times. From the secondary literature, we added another study published in 2018.

### Intensive training to implement the 2 h limit

Intensive training can reduce liquid fasting time to some extent. In two recent studies, liquid fasting time before surgery was significantly reduced by education/training interventions. However, the fasting times achieved were still significantly longer than those recommended by the guidelines, at >5 h.^129^^,^^130^ The introduction of a fasting guideline reminder via a mobile phone SMS by Zia and colleagues^131^ in addition to a written hospital policy reduced median liquid fasting times by 3 h, but only 13.6% of patients fasted appropriately. Komatsu and colleagues^132^ reported a reduction in liquid fasting time from 243 to 180 min through multidisciplinary interventions in a perioperative management centre. However, only 8% of patients consumed >200 ml of clear liquid at baseline and at the end of the study.

### Different approaches to implement ‘Sip Til Send’

In 2021, Sands and colleagues^141^ submitted freedom of information requests to all acute National Health Service England Hospital Trusts. The results revealed that 21 out of 100 Trusts now have preoperative intake guidelines that allow water after the 2-h cut-off recommended by current national guidance and 15 Trusts allow water to be sipped up to the point of sending for the patient for theatre. None of these Trusts are reporting increased rates of adverse events in the current literature or safety publications.^141^

In some centres, only drinking water is allowed. In the UK, Kannan^140^ started a ‘THINK DRINK’ campaign in which, on the day of arrival, surgical patients get a ‘welcome drink’ of water on admission and are allowed unlimited sips of water until being called. Patients showed a reduction in the mean fasting time for liquids to 2 h. Daly and colleagues^100^ allowed one glass of water (160 ml) per hour for women undergoing elective Caesarean delivery under spinal anaesthesia. Liquid fasting time was reduced from almost 8 h to 55 min with significant reductions in thirst, nausea, discomfort, light-headedness, and anxiety. However, in a study by Bouvet and colleagues^148^ allowing only limited water consumption does not ensure high patient satisfaction, at least during delivery. The authors concluded that fresh clear liquids, unrestricted amounts of liquids and sweet liquids could improve patient comfort.

In one British study and one Dutch study, all clear liquids were allowed; however, the amount that could be consumed was limited. The ‘Sip Til Send’ policy implemented by Checketts,^142^ allowing one 170 ml glass of clear liquid per hour until patients are sent for their procedure, reduced liquid fasting times from 6 h to 17 min. They did not observe an increase in reported adverse events in >12 000 patients through ongoing governance monitoring. The Dutch study by Marsman and colleagues^52^ reduced the median fasting time to 74 min by allowing clear liquids to be consumed until arrival in the operating theatre, with a maximum of 1 glass per hour and an additional glass with premedication (see below for more details).

In two studies, patients were allowed to drink all clear liquids without restrictions on the amount. In the UK, McCracken and Montgomery^85^ allowed >4700 day case patients unrestricted clear oral fluids before operation until transfer to the theatre. They demonstrated a significant reduction in nausea without adverse events of pulmonary aspiration of gastric contents requiring hospital admission. A German quality improvement study used fasting cards to implement unrestricted drinking of clear liquids until called to the theatre. Using this approach, they reduced the median liquid fasting time from 12 to 2.1 h.^83^ Patients who were allowed unrestricted drinking before surgery drank wisely and according to their needs.^149^ Neither sonographically nor gastroscopically did the ‘Sip Til Send’ regimen result in a clinically relevant increase in residual gastric volume.^150^^,^^151^

## Learning points

• In children, liberal liquid fasting regimens even below the recommended 1 h are much more common than in adults.
• In adults, quality improvement measures have not yet resulted in liquid fasting times in the range of the recommendation of 2 h.
• When drinking was allowed within 2 h before induction of anaesthesia, there was a significant reduction in fasting times for liquids.

## Perspectives

However, no adequately powered studies have demonstrated the safety of short liquid fasting times in patients, particularly with regard to the risk of aspiration. The authors of the updated American guideline have calculated that, with a baseline incidence of 1.1/10 000 cases of aspiration in elective patients, 214 000 participants per study arm would be required in a two-arm study to demonstrate a two-fold increase (power, 80%; α, 0.05).^79^ So far, no study has met this requirement for the number of cases. However, the data from Marsman and colleagues^52^ are a first step.

In 2023, Marsman and colleagues^52^ achieved a median liquid fasting time of 74 min in 16 815 patients with a liberal liquid fasting policy (for details see above), with no significant increase in the incidence of regurgitation or aspiration compared with 59 636 control patients who followed standard fasting rules. Four patients of the liberal fasting group aspirated; three of them developed an aspiration pneumonia. One of these patients had a liquid fasting time of 2.09 h, one aspirated food, and in one patient an ileus was missed. Patients with regurgitation (*n*=146) had a mean liquid fasting duration of 2.52 h compared with patients without regurgitation (*n*=76 305) with a liquid fasting duration of 2.36 h.

### What is coming next?

• With the new upcoming guideline ‘Perioperative Fasting in Adults’, a systematic literature review from 2010 onwards on the impact of preoperative fasting on perioperative outcomes is expected, together with a revision of the recommendations.^77^
• Eurofast, a large multicentre study, will monitor the incidence of pulmonary aspiration in 180 000 paediatric patients in relation to liquid fasting time. The study is expected to be completed by the end of June 2024.^152^
• A multicentre randomised controlled study on ‘Preoperative Fasting Versus Not Fasting in Critically Ill Patients’.^153^
• An ongoing systematic review on ‘Abbreviation of preoperative fasting in surgical patients’ is analysing articles published in the last 10 yr and is expected to be completed by the end of February 2024.^154^

## Learning points

• Adequately powered studies demonstrating the safety of liberal liquid fasting regimes in patients, particularly regarding the risk of aspiration, are lacking.

## Conclusion

Attempts to reduce fasting times through quality improvement measures have failed so far. Concepts such as ‘Sip Til Send’ have achieved significant reductions in fasting times and improvements in patient well-being. However, these concepts need to be further investigated in well-designed, large clinical trials to assess patient safety, focusing on both the risk of aspiration and the complications of prolonged fasting.

## Authors’ contributions

Conception and interpretation of this work: all authors

Drafting of the manuscript: AR

Critical revisions of the manuscript: PM, EN

Approved the ﬁnal version of the manuscript: all authors

Accountable for all aspects of the work: all authors

## Declarations of interest

The authors declare that they have no conﬂicts of interest.
